# Beyond connectedness: why pairwise metrics cannot capture community stability

**DOI:** 10.1002/ece3.2461

**Published:** 2016-09-16

**Authors:** Anje‐Margriet Neutel, Michael A. S. Thorne

**Affiliations:** ^1^ British Antarctic Survey Cambridge UK

**Keywords:** connectance, ecological networks, feedback loops, food webs, interaction strength, stability

## Abstract

The connectedness of species in a trophic web has long been a key structural characteristic for both theoreticians and empiricists in their understanding of community stability. In the past decades, there has been a shift from focussing on determining the number of interactions to taking into account their relative strengths. The question is: How do the strengths of the interactions determine the stability of a community? Recently, a metric has been proposed which compares the stability of observed communities in terms of the strength of three‐ and two‐link feedback loops (cycles of interaction strengths). However, it has also been suggested that we do not need to go beyond the pairwise structure of interactions to capture stability. Here, we directly compare the performance of the feedback and pairwise metrics. Using observed food‐web structures, we show that the pairwise metric does not work as a comparator of stability and is many orders of magnitude away from the actual stability values. We argue that metrics based on pairwise‐strength information cannot capture the complex organization of strong and weak links in a community, which is essential for system stability.

## 1 Introduction

One of the central challenges for ecologists is to understand the relation between the structure and stability of ecological communities. The traditional idea that the multitude of interactions in a community contribute to its stability (Elton, 1927; MacArthur, 1955; Odum, 1971) was challenged when ecologists started using models of dynamical systems, and it was shown that communities where species were more connected were less likely to be stable (Gardner & Asby, 1970; May, 1972). However, in the past decades it has become increasingly clear that the focus on the number of interactions is too limited and that the patterning of strong and weak interactions in communities is crucial to its stability (Banašek‐Richter, Cattin, & Bersier, 2004; Banašek‐Richter et al., 2009; Bersier, Banašek‐Richter, & Cattin, 2002; Brose, Williams, & Martinez, 2006; Drossel, McKane, & Quince, 2004; Emmerson & Raffaelli, 2004; Gross, Rudolf, Levin, & Dieckmann, 2009; James et al., 2015; Kondoh, 2003; McCann, Hastings, & Huxel, 1998; Mitchell & Neutel, 2012; Montoya, Woodward, Emmerson, & Solé, 2009; Neutel, Heesterbeek, & de Ruiter, 2002; Neutel & Thorne, 2014; Neutel et al., 2007; Novak et al., 2011; Paine, 1988, 1992; Polis & Strong, 1996; Rooney, McCann, Gellner, & Moore, 2006; de Ruiter, Neutel, & Moore, 1995; Ulanowicz, Holt, & Barfield, 2014; Wootton, 1994; Wootton & Emmerson, 2005; Yodzis, 1981). May's random matrix theorem (May, 1972), which distinguishes the number of species, their connectance (number of possible interactions that are realized), and average interaction strength, is not able to explain local stability of community models with interaction strengths (sensu May, 1972) parameterized from observation (Jacquet et al., 2016; James et al., 2015; Neutel & Thorne, 2014; Neutel et al., 2002, 2007). Various studies have suggested alternative connectance measures which incorporate the strength of interactions (Banašek‐Richter et al., 2009; Bersier et al., 2002; Ulanowicz, 1997; Van Altena, Hemerik, & de Ruiter, 2016). Furthermore, it has been argued that in order to understand community stability, we need to look at the feedback structure formed by the interactions (Levins, 1974) and quantify critical feedback loops (Neutel et al., 2002). Feedback loops are cycles of causal effects (Levins, 1974). In particular, the strength of three‐link loops has been shown to be key to stability (Mitchell & Neutel, 2012; Neutel et al., 2007), and recently, a metric has been proposed that compares the stability of observed trophic networks in terms of the strength of the three‐link relative to two‐link feedback loops in a system (Neutel & Thorne, 2014). The two‐link loops in trophic networks represent the product of the effect of a predator on a prey (negative link) and the reciprocal effect of this prey on its predator (positive link). The three‐link loops are loops in the smallest omnivorous structures [also called intraguild predation (Holt & Huxel, 2007), between a predator, an intermediate prey–predator, and the shared prey of this intermediate and its predator]. These structures form positive (one positive and two negative links) and negative (one negative and two positive links) three‐link loops, quantified as products of the three effects (Levins, 1974). The understanding in terms of key feedback loops has revealed that it is not network complexity (the number of species or their connectance) which puts constraints on system stability, but the energy‐flow and biomass distribution in the trophic pyramid (Neutel et al., 2002). Specifically, it has shown how increased predation pressure over trophic levels leads to less stability (Neutel & Thorne, 2014; Neutel et al., 2007).

However, it has also been suggested that in order to capture the stability of empirical trophic networks, one does not need to go beyond the pairwise interactions (Smith, Sander, Barabás, & Allesina, 2015; Tang, Pawar, & Allesina, 2014). Commenting on Neutel and Thorne (2014), Smith et al. (2015) argue that the metric proposed by Tang et al. (2014), which is based on random matrix theory (Allesina & Tang, 2012; May, 1972) and uses pairwise information of the interaction strengths, in the form of the correlation between effects of predators on prey and vice versa, will be a better estimator of stability. They imply that the match between the feedback metric and system stability found by Neutel and Thorne (2014) depends on the particular data set, obtained using a scaling procedure to make the interaction strengths dimensionless.

Here, we compare the ability of these two recently proposed metrics to explain local stability of the observed food‐web structures studied by Neutel and Thorne (2014). First, we take the original, observed interaction strengths (Jacobian matrix elements, sensu May (1972); see also Berlow et al., 2004, table 1) and apply the feedback metric and the pairwise metric to these data. We then apply both metrics to the scaled data set used by Neutel and Thorne (2014). The scaling was introduced by Neutel and Thorne (2014) to translate the observed structure in the intraspecific interaction strengths (diagonal matrix elements) into the off‐diagonal structure, in order to analyze the feedback structure without losing the intraspecific information. Next, we parameterize the same observed food‐web structures with synthetic interaction strengths, first with an asymmetry within predator–prey pairs of interaction strengths, and then with completely random (symmetric) strengths, to investigate to what extent the results depended on the empirical parameterizations. Finally, we perform a disturbance experiment with the empirical data, in the example of Yodzis (1981), where we disrupt the empirical patterning of interaction strengths by randomly swapping pairs of predator–prey interaction strengths in the matrices. We do this to show to what extent the metrics are able to explain the impact of the empirical organization of interaction strengths on community stability.

## 2 Methods

### 2.1 Empirical food webs

Our local stability analysis was performed on Jacobian community matrix models (linearizations of systems of differential equations) of the same food‐web structures as were used for the feedback analysis in Neutel and Thorne (2014). These were 23 observed food webs (Neutel & Thorne, 2014, table S3): two Antarctic food webs with interaction strengths quantified from independent flux observations (Neutel & Thorne, 2014, 2016b) and 21 soil food webs with interaction strengths quantified from inferred fluxes based on time‐averaged biomass observations (de Ruiter et al., 1995; de Ruiter, Neutel and Moore 2016; Neutel et al., 2007, 2016). Our analysis was on the 23 trophic networks (+/− structure, consumer–resource, or predator–prey interactions) in these food webs, obtained by removing the detritus row and column from the matrices.

### 2.2 Underlying dynamics of the populations

The fluxes between the populations *X*_*i*_ (with *X* referring to its biomass) of each population *i* = 1 ··· *n* in a food web were described by:

(1) $$\frac{dX_{i}}{dt}=r_{i}X_{i}-m_{i}X_{i}+\sum_{h=1}^{n}e_{hi}f_{i}\left(X_{h}\right)X_{i}-\sum_{j=1}^{n}f_{j}\left(X_{i}\right)X_{j}-c_{ii}X_{i}^{2},\;i=1\ldots n$$

where *f*_*i*_(*X*_*h*_) is the functional response in a consumer, *r*_*i*_ is the intrinsic growth rate, *m*_*i*_ is the intrinsic loss rate (we assume net intrinsic growth for basal species and *r*_*i*_ **= 0 for nonbasal species), *c*_*ii*_ is a proportionality constant referring to intraspecific competition, and *e*_*hi*_ is a biomass conversion efficiency.

We assumed linear functional responses in this study (following Neutel & Thorne, 2014). However, the community matrices with underlying linear functional responses can be easily translated into ones based on nonlinear responses, and the results are robust to these other types of functional response (see Neutel & Thorne, 2016a). Assuming linear functional responses, equation (1) becomes a Lotka–Volterra‐type equation with intraspecific competition terms (see, e.g., Pimm, 1982):

(2) $$\frac{dX_{i}}{dt}=r_{i}X_{i}-m_{i}X_{i}+\sum_{h=1}^{n}e_{hi}c_{hi}X_{h}X_{i}-\sum_{j=1}^{n}c_{ij}X_{i}X_{j}-c_{ii}X_{i}^{2},\;i=1\ldots .n$$

### 2.3 Parameterization of Jacobian community matrices

The interaction strengths between the populations are the elements of the Jacobian community matrix, a linearization of the system around the nontrivial equilibrium (where each species has a positive population density). Thus, they are the partial derivatives of the population growth equations (dimension per time) evaluated at equilibrium (May, 1973). Using equation (2), the elements of the community matrix ***Α*** are effects of predator *j* on prey *i*, $\alpha_{ij}=-c_{ij}X_{i}^{}*$ ; effects of prey *h* on consumer *i*, $\alpha_{ih}=e_{hi}c_{hi}X_{i}^{*}$; and intraspecific effects, $\alpha_{ii}=-c_{ii}X_{i}^{}*$ (because at equilibrium, $r_{i}-m_{i}+\sum_{h=1}^{n}e_{hi}c_{hi}X_{h}-\sum_{j=1}^{n}c_{ij}X_{j}-c_{ii}X_{i}=0)$. Thus, the negative effects of predators on prey are the feeding rates of a predator on its prey divided by predator biomass, and the positive effects of prey on their predators are predator growth rates divided by prey biomass (Pimm, 1982; de Ruiter et al., 1995; Yodzis, 1989).

The interspecific interaction strengths of the Antarctic food webs were quantified from direct flux observations (Neutel & Thorne, 2014, 2016b), and those of the 21 soil food webs were quantified from inferred fluxes based on time‐averaged biomass observations (de Ruiter et al., 1995; de Ruiter et al. 2016; Neutel et al., 2007, 2016). The intraspecific interaction strengths (diagonal elements) were not obtained from directly observed fluxes, but the observations provided upper bounds. The rationale is as follows: For a given food web in equilibrium, total loss for each population equals total gain. Growth rates and predatory loss rates of each population were known; hence, nonpredatory loss rates were also known, because the systems were in equilibrium. This total nonpredatory loss rate consists of intrinsic death and intraspecific competition: $d_{i}X_{i}^{*}=m_{i}X_{i}^{*}+c_{ii}X_{i}^{*2}$ . The amount of intraspecific competition is hence contained within the energetic boundaries of the system $0\geq c_{ii}X_{i}^{*}\geq d_{i}$ . The upper bounds were used to quantify the diagonal elements of (un‐normalized) community matrix ***Α***: $\alpha_{ii}=-c_{ii}X_{i}^{*}=-d_{i}$ .

### 2.4 Scaled interaction strengths

Following Neutel & Thorne, 2014; interaction strengths were scaled by dividing each row in community matrix ***Α*** by the absolute value of its respective diagonal element, $\alpha_{ij}/\left|\alpha_{ii}\right|$, which resulted in time‐independent and dimensionless matrices ***Γ***. This scaling procedure was introduced by Neutel and Thorne (2014) to translate the diagonal structure of the matrix into the off‐diagonal structure, and obtain an eigenvalue which, for their observed food webs, was equivalent to a critical value of intraspecific competition for stability [specifically, it represents the proportion of total nonpredatory loss needed for stability; see Neutel et al. (2002)].

### 2.5 Determination of stability

The diagonal elements of the Jacobian and scaled matrices were then set at zero, obtaining matrices ***Α***_***0***_ and ***Γ***_***0***_. System stability was determined as the largest real part of the eigenvalues of these matrices, *λ*_*d*_. By definition, with all diagonal elements set at zero, then *λ*_*d*_ ≥ 0; that is, the systems need some level of self‐damping in order to be stable, and −*λ*_*d*_ is the amount of self‐damping needed for stability. In the case of the normalized matrix **Γ**_**0**_, *λ*_*d*_ has a biological meaning and indicates a tipping point. It represents a critical level of intraspecific competition of the populations as a proportion of the maximum possible intraspecific competition (the upper bound of the diagonal elements) (Neutel & Thorne, 2014). In the case of the Jacobian matrix **Α**_**0**_, *λ*_*d*_ is related to the timescales of the systems and is not easily interpretable biologically given the different intraspecific interaction strengths of the populations. It is the opposite of a system's resilience. The inverse of *λ*_*d*_ of **Α**_**0**_ is the time with which the system moves away from the equilibrium, after a very small disturbance.

### 2.6 Synthetic parameterization of community matrices

For the synthetic parameterization of the community matrices, the empirical values of the nonzero off‐diagonal elements were first replaced by values randomly drawn from uniform distributions (−10, 0) for effects of predators on prey and (0, 0.1) for effects of prey on predators (following Pimm & Lawton, 1978; see also Neutel & Thorne, 2014). This procedure was then repeated without the asymmetry in size ranges, drawing values from uniform distributions (−1, 0) and (0, 1) (following May, 1972; see also Neutel & Thorne, 2014).

### 2.7 Pairwise disturbance experiment

To show the effect of the patterning of interaction strengths on stability, we performed a disturbance experiment, following Yodzis (1981). For each empirical community matrix **Α**_**0**_ **,** the pairs of nonzero off‐diagonal elements were randomly permuted. This preserved the sign structure and the pairwise structure of the interaction strengths of the original matrix.

### 2.8 Feedback metric

The metric proposed by Neutel and Thorne (2014) expresses a ratio between three‐link and two‐link feedback: $\sqrt[{3}]{\left\lfloor \frac{a_{3}}{a_{2}}\right\rfloor }$, where *a*_*2*_ and *a*_*3*_ are coefficients of the characteristic polynomial of the community matrix. In a matrix with zero‐diagonal elements, the second coefficient, *a*_*2*_, represents the sum of all the two‐link feedback loops (*F*_*2*_), resulting from the pairs of predator–prey interactions, which are by definition negative: In community matrix **Α**_**0**_, $F_{2}=\sum \alpha_{ij}\alpha_{ji}$ (the same holds for normalized matrix **Γ**_**0**_). The third coefficient, *a*_*3*,_ in zero‐diagonal matrices, represents the sum of all the three‐link loops (*F*_*3*_), coming from the smallest omnivorous structures, each generating a positive and a counteracting negative feedback loop: $F_{3}=\sum \left(\alpha_{ij}\alpha_{jk}\alpha_{ki}+\alpha_{ik}\alpha_{kj}\alpha_{ji}\right)$ , where *i* is the bottom prey, *j* is the intermediate predator, and *k* is the omnivore. The sum of the positive and counteracting negative loop in each three‐link omnivorous structure is by definition positive, given the functional assumptions (Neutel & Thorne, 2014).

### 2.9 Pairwise metric

The metric proposed by Tang et al. (2014) quantifies the overall correlation between effects of predators on prey and vice versa: $\sqrt{SV}\left(1+\rho \right)-E$, where *S* is the number of “species,” *E* is the mean of the off‐diagonal elements of the community matrix, *V* is their variance, and *ρ* is the overall pairwise correlation between the elements of the community matrix (*α*_*ij*_, *α*_*ji*_)_*i*≠*j*_ (Tang et al., 2014).

## 3 Results

**Figure 1:**
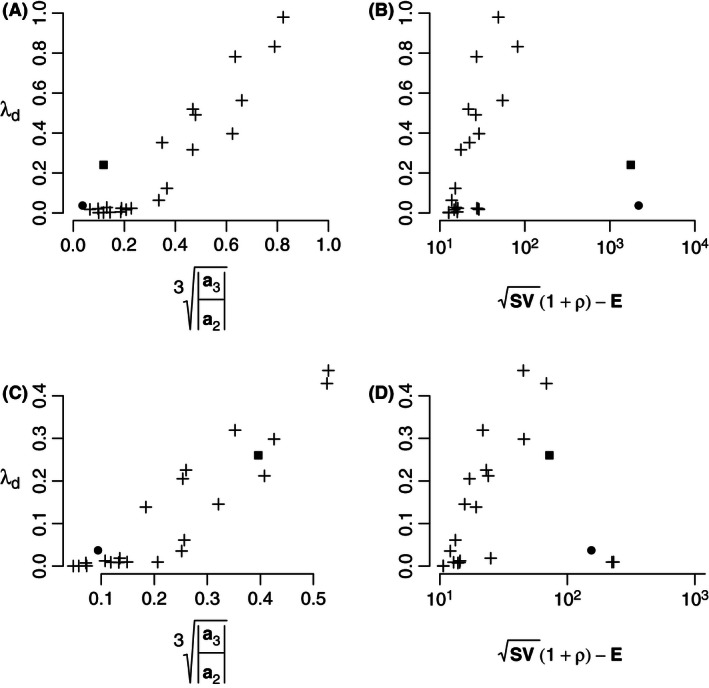
Comparison of the performance of Neutel and Thorne's feedback metric $\sqrt[{3}]{\left|a_{3}\right|/\left|a_{2}\right|}$ (Neutel & Thorne, 2014) with Tang et al.'s pairwise metric $\sqrt{SV}\left(1+\rho \right)-E$ (Tang et al., 2014) across ecosystems, for empirically parameterized community matrices. For an explanation of the metrics, see Methods. Stability (*λ*_*d*_) of the Antarctic dry (closed circle) and wet (closed square) tundra ecosystems and 21 soil food webs (cross signs) (see Neutel & Thorne, 2014, table S3; de Ruiter, Neutel, & Moore, 2016; Neutel & Thorne, 2016b; Neutel et al., 2016). (A, B) Original interaction strengths (elements of Jacobian community matrix **Α**_**0**_, with dimension *t* ^−1^). (C, D) Scaled interaction strengths (elements of scaled community matrix **Γ**_**0,**_ dimensionless**)**. (A: *N* = 23, adjusted *R* ^2^ = .87, *p* < 10^−9^; B: *N* = 23, *R* ^2^ = −.02, *p* = .48; C: *N* = 23, *R* ^2^ = .84, *p* < 10^−9^; D: *N* = 23, *R* ^2^ = .004, *p* = .31.) Note that all diagonal elements were set at zero

We found a strong correlation between our feedback metric and stability for the 23 empirical food webs parameterized with the original interaction strengths (observed Jacobian community matrices) (Fig. 1A). The pairwise metric showed no relation with food‐web stability, neither for the original, nor for the scaled interaction strengths, and underestimated the stability by many orders of magnitude (Fig. 1B and D), while the feedback metric explained both data sets equally well (Fig. 1A and C).

**Figure 2:**
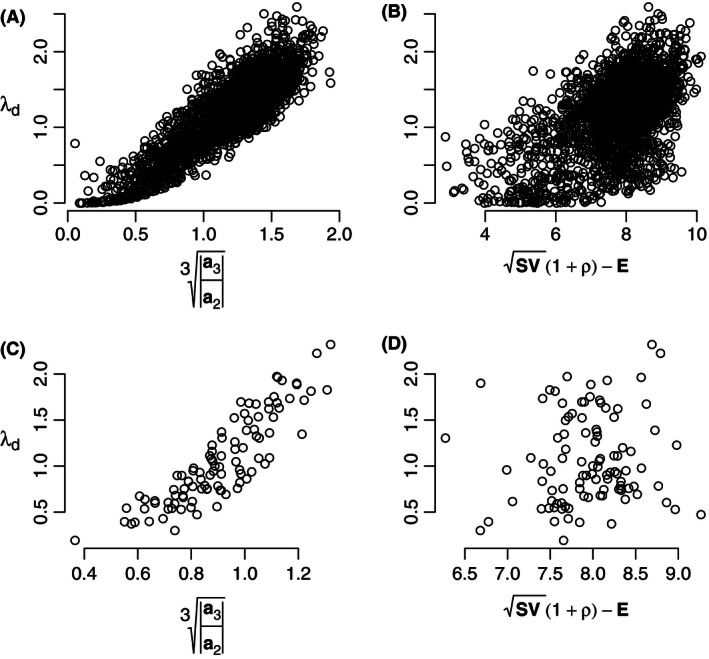
Comparison as in Fig. 1, for synthetic, asymmetric parameterizations of the interaction strengths. (A, B) Food‐web structures as in Fig. 1, but with nonzero matrix element values randomly drawn from asymmetric intervals (−10, 0) and (0, 0.1) (following Pimm & Lawton, 1978), for 100 individual samplings of each of the 23 food webs. (C, D) As in (A) and (B), but on a single network, using the food‐web structure of the Antarctic dry tundra ecosystem, from Neutel and Thorne (2014) (A: *N* = 2300, *R* ^2^ = .76, *p* < 10^−15^; B: *N* = 2300, *R* ^2^ = .30, *p* < 10^−15^; C: *N* = 100, *R* ^2^ = .77, *p* < 10^−15^; D: *N* = 100, *R* ^2^ = −.0026, *p* = .39)

When we parameterized the same interactions in these food webs with random‐type values, imposing a simple asymmetry between negative and positive interaction strengths (Pimm & Lawton, 1978; see also Neutel & Thorne, 2014), both metrics showed a relation with food‐web stability, but the feedback metric outperformed the pairwise metric (Fig. 2A and B). We then calculated system stability for different parameterizations (sampling from the two size distributions, as above) of the same food‐web structure. Thus, we removed the effect of system size and connectance on stability. This made clear that the pairwise metric was not able to explain the relation between the structure and stability of a system, in contrast to the feedback metric (Fig. 2C and D).

**Figure 3:**
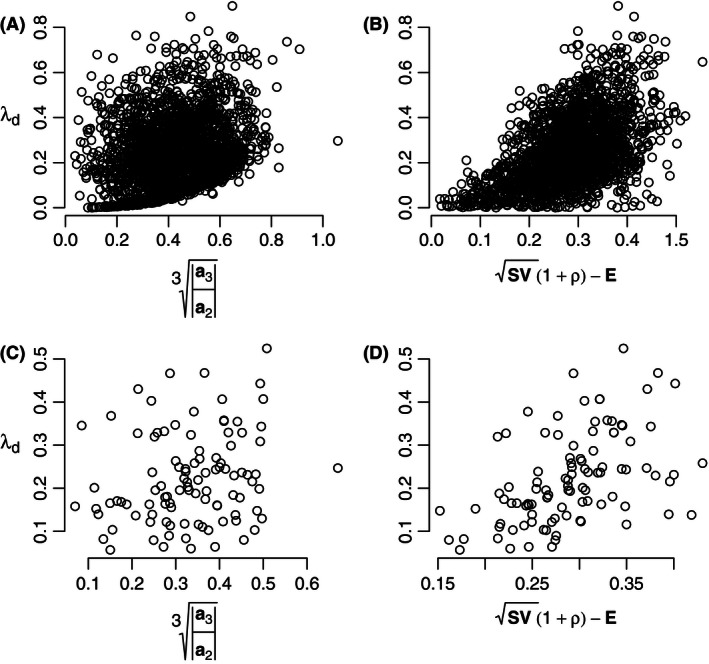
Comparison as in Fig. 1, for synthetic, symmetric parameterizations of the interaction strengths. (A, B) Food‐web structures as in Fig. 1, but with nonzero matrix element values randomly drawn from symmetric intervals (−1, 0) and (0, 1) (following May, 1972), for 100 individual samplings of each of the 23 food webs. (C, D) As in (A) and (B), but on a single network, using the food‐web structure of the Antarctic dry tundra ecosystem, from Neutel and Thorne (2014) (A: *N* = 2300, *R* ^2^ = .046, *p* < 10^−15^; B: *N* = 2300, *R* ^2^ = .26, *p* < 10^−15^. C: *N* = 100, *R* ^2^ = .03, *p* = .038; D: *N* = 100, *R* ^2^ = .22, *p* < 10^−6^)

Next, we repeated this procedure, using a symmetry between negative and positive interaction strengths (following May, 1972; see also Neutel & Thorne, 2014). For these random parameterizations drawn from symmetric intervals, the feedback metric did not show any relation with food‐web stability, neither for the 23 webs, nor for different parameterizations of a single web structure (Fig. 3A and C), while the pairwise metric showed some correlation (Fig. 3B and D).

**Figure 4:**
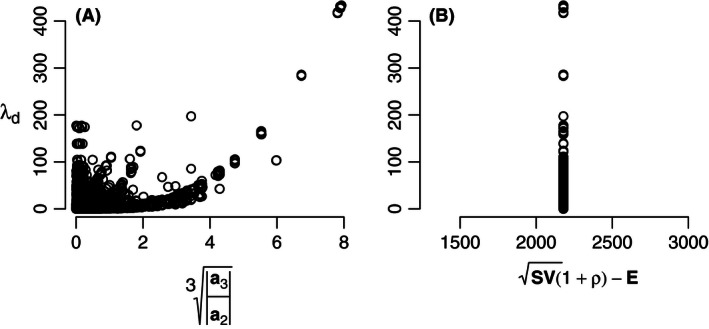
Stability (*λ*_*d*_) after disruption of the empirical patterning of interaction strengths by random permutation of the nonzero matrix element pairs $\left(\alpha_{ij},\alpha_{ji}\right)$ of **Α**_**0**_ (following Yodzis, 1981), using the Jacobian matrix of the Antarctic dry tundra ecosystem (for stability of the intact pattern, see closed circle in Fig. 1A,B). (A) Feedback metric $\sqrt[{3}]{\left|a_{3}\right|/\left|a_{2}\right|}$ (Neutel & Thorne, 2014) (A: *N* = 10^5^, *R* ^2^ = .38, *p* < 10^−15^) and (B) the pairwise metric $\sqrt{SV}\left(1+\rho \right)-E$ (Tang et al., 2014). Note that in these randomly permuted webs, the topology, sign structure, and pairwise structure of the empirical food web stayed intact

Finally, we analyzed the effect of the empirical organization of the interaction strength values on stability, by disturbing this organization through randomly permuting pairs of interaction strengths (following Yodzis, 1981). The feedback metric was unable to explain the resulting loss of organization in a satisfactory way, although it did show some correlation with stability, within a diversity of pattern (Fig. 4A). However, by definition the pairwise metric could not explain any difference in organization (Fig. 4B).

## 4 Discussion

The importance of the patterning of weak and strong interactions for the stability of ecological communities (McCann et al., 1998; Neutel et al., 2002; Paine, 1988, 1992; Polis & Strong, 1996; de Ruiter et al., 1995; Wootton, 1994; Yodzis, 1981) merits the question of how much of the organization of interaction strengths has to be taken into account to capture community stability. Smith et al. (2015) argue that the metric proposed by Tang et al. (2014), which quantifies pairwise connectedness, will provide a better estimate of food‐web stability than the metric proposed by Neutel and Thorne (2014), which quantifies three‐link and two‐link feedback loops and imply that we do not need to go beyond the pairwise interaction strengths, to explain the stability of complex natural communities.

Our results show, however, that Tang et al.'s metric does not explain the stability of models parameterized with empirical data. For food‐web structures in the size range for which empirical data are available, Neutel and Thorne's feedback metric (Neutel & Thorne, 2014) is a good comparator of stability, whether the interaction strengths are scaled, as analyzed by Neutel and Thorne (2014), or not. Furthermore, even for synthetic parameterizations with an asymmetry between effects of predators on prey and vice versa, the feedback metric is a better comparator and estimator of stability than the pairwise metric. This is remarkable, because the pairwise metric has been particularly aimed at dealing with this type of synthetic parameterizations (Tang et al., 2014). Our application of both metrics to different parameterizations of a single food‐web structure shows that the pairwise metric is not able to capture the structure of a system (Fig. 2C). The correlation between the pairwise metric and comparative stability of the different food‐web structures is an artifact of the synthetic parameter values—the metric effectively measures system size. Furthermore, this comparison of different synthetic asymmetric parameterizations of a single web (Fig. 2C) also shows that Neutel and Thorne's feedback metric cannot be approximated by a connectance‐based analogue, such as suggested by Smith et al. (2015). Their approximation is a simplification of the feedback metric resulting from assumptions on random parameter values. It cannot, by definition, explain the differences in stability shown in Fig. 2C, because all these model samples share the same connectance.

Only parameterizations sampled randomly from symmetric intervals for predator–prey and prey–predator effects are not captured by the feedback metric. This confirms the findings of Neutel and Thorne (2014), who show that it is the dominance of positive feedback in the three‐link loops, which underlies the relation between the feedback metric and system stability. As they show, this dominance of positive feedback is brought about by the well‐known asymmetry within pairs of predator–prey interaction strengths (see Pimm & Lawton, 1978 and de Ruiter et al., 1995).

When we performed a pairwise disturbance of the empirically parameterized food webs (following Yodzis, 1981), the feedback metric, while showing some correlation within a diversity of pattern, was unable to explain the effects on stability in a satisfactory way, indicating that more understanding is needed. However, pairwise metrics do not provide an alternative, because they cannot capture the complex organization of strong and weak links in a trophic network. It is logically impossible for any pairwise metric (Allesina & Tang, 2012; May, 1972; Tang et al., 2014) to explain the effect on stability of a pairwise disturbance experiment (Yodzis, 1981). For a better understanding of how organization affects stability, it may be necessary to look at the spectrum of strengths of three‐ and two‐link loops, instead of just the total strength of three‐link versus two‐link loops (see Neutel & Thorne, 2014; Neutel et al., 2002).

Quantifying the feedback structure of ecological networks is not only necessary to compare the stability of ecological systems, but also provides a way forward to understand the underlying assumptions on the functionality and adaptive strategies of populations (Neutel & Thorne, 2016a).

It should be emphasized that the empirical data on biomass and energy flow for each of the food webs in this study are of the highest quality available, and were obtained not with one specific method for all the webs, but with different methodologies (Neutel & Thorne, 2014). At present, such data only exist for systems in the size range presented here, of 10–30 trophic groups. There is a pressing need to obtain realistic, empirical, data for a wider and larger range of systems, to test the feedback metric and, if needed, make further improvements. This heuristic approach will prevent us from being wrong‐footed by an artificial generality brought about by a random parameter space (Neutel & Thorne, 2014). It is already clear from the existing empirical evidence, however, that in order to capture the “organized complexity” of communities which characterizes the functioning of real ecosystems, the only way forward is to take the step beyond pairwise interactions.

## Funding Information

Natural Environment Research Council.

## Conflict of Interest

None declared.
